# Cytokinin is required for escape but not release from auxin mediated apical dominance

**DOI:** 10.1111/tpj.12862

**Published:** 2015-05-12

**Authors:** Dörte Müller, Tanya Waldie, Kaori Miyawaki, Jennifer PC To, Charles W Melnyk, Joseph J Kieber, Tatsuo Kakimoto, Ottoline Leyser

**Affiliations:** 1 Department of Biology, University of YorkHeslington, York, YO10 5DD, UK; 2 Sainsbury Laboratory, University of CambridgeBateman Street, Cambridge, CB2 1LR, UK; 3 Biology Department, University of North CarolinaChapel Hill, NC, 27599, USA; 4 Department of Biological Sciences, Graduate School of Science, Osaka UniversityMachikaneyama, Toyonaka, Osaka, 560-0043, Japan; Shanghai Center for Plant Stress Biology3888 Chenhua Road, Shanghai, 201602, China

**Keywords:** cytokinin, auxin, shoot branching, apical dominance, *Arabidopsis thaliana*, Isopentenyltransferase, type-A Arabidopsis response regulators

## Abstract

Auxin produced by an active primary shoot apex is transported down the main stem and inhibits the growth of the axillary buds below it, contributing to apical dominance. Here we use *Arabidopsis thaliana* cytokinin (CK) biosynthetic and signalling mutants to probe the role of CK in this process. It is well established that bud outgrowth is promoted by CK, and that CK synthesis is inhibited by auxin, leading to the hypothesis that release from apical dominance relies on an increased supply of CK to buds. Our data confirm that decapitation induces the expression of at least one ISOPENTENYLTRANSFERASE (*IPT*) CK biosynthetic gene in the stem. We further show that transcript abundance of a clade of the CK-responsive type-A Arabidopsis response regulator (*ARR*) genes increases in buds following CK supply, and that, contrary to their typical action as inhibitors of CK signalling, these genes are required for CK-mediated bud activation. However, analysis of the relevant *arr* and *ipt* multiple mutants demonstrates that defects in bud CK response do not affect auxin-mediated bud inhibition, and increased *IPT* transcript levels are not needed for bud release following decapitation. Instead, our data suggest that CK acts to overcome auxin-mediated bud inhibition, allowing buds to escape apical dominance under favourable conditions, such as high nitrate availability.

**Significance Statement:**

It has been proposed that the release of buds from auxin-mediated apical dominance following decapitation requires increased cytokinin biosynthesis and consequent increases in cytokinin supply to buds. Here we show that in Arabidopsis, increases in cytokinin appear to be unnecessary for the release of buds from apical dominance, but rather allow buds to escape the inhibitory effect of apical auxin, thereby promoting bud activation in favourable growth conditions.

## Introduction

Branches develop from axillary meristems (AMs), established in the axils of leaves made by the primary shoot apical meristem (SAM). Axillary meristems typically produce a few leaves before entering a dormant state as an axillary bud. The degree of branching is determined by the subsequent reactivation of these buds. Branching impacts upon light harvest, biomass and seed yield, but must be modulated by the availability of resources and competing growth requirements in the roots. The ability of plants to regulate bud outgrowth in response to environmental parameters is therefore of considerable adaptive significance and must be finely tuned. This is achieved in part by a network of hormones, which together integrate environmental and endogenous inputs. A central player is auxin, an inhibitor of bud outgrowth, which is synthesised in young leaves (Ljung *et al*., 2001) and transported rootward in the polar auxin transport stream (PATS). Apical decapitation, removing the main auxin source, results in the sustained outgrowth of buds in the axils of leaves below, which can be prevented by applying auxin to the decapitation site (Thimann and Skoog, 1933). Radio-label experiments have demonstrated that auxin acts indirectly, as it does not enter buds in significant amounts (Hall and Hillman, 1975; Morris, 1977; Prasad *et al*., 1993; Booker *et al*., 2003; Petrášek and Friml, 2009).

Two general mechanisms for indirect inhibition by auxin have been proposed. Firstly, the canalisation-based hypothesis states that for a bud to activate it must establish its own PATS into the main stem (Prusinkiewicz *et al*., 2009). Activation is prevented by high auxin in the main stem, making it a weak sink for auxin from the bud (Sachs, 1981, 2000). In this model, shoot apices compete for access to the main stem PATS. The second hypothesis proposes that auxin affects the level of a second messenger, which moves into the bud and regulates bud activity. Strigolactone (SL) and cytokinin (CK) are both good candidates for this second messenger. Strigolactone biosynthetic genes are upregulated by auxin in the main stem (Sorefan *et al*., 2003; Foo *et al*., 2005; Johnson *et al*., 2006; Zou *et al*., 2006; Arite *et al*., 2007; Hayward *et al*., 2009; Zhang *et al*., 2010; Waters *et al*., 2012) and direct application of SL to buds can inhibit their activity (Gomez-Roldan *et al*., 2008; Brewer *et al*., 2009; Hamiaux *et al*., 2012). In contrast, CK biosynthetic genes are downregulated by auxin in the main stem (Tanaka *et al*., 2006) and direct application of CK to buds causes activation *(*Wickson and Thimann, 1958; Pillay and Railton, 1983; Cline *et al*., 1997). In pea, there is a strong correlation between bud outgrowth and the CK:SL ratio in buds, evidenced using exogenous hormone application and SL mutant analysis. This is proposed to be mediated by the opposite effects of SL and CK on the expression of *BRANCHED1* (Braun *et al*., 2012; Dun *et al*., 2012), a member of the *TEOSINTE BRANCHED1*,*CYCLOIDEA* and *PROLIFERATING CELL FACTORS 1* and *-2* (*TCP*) gene family with a well-supported role in inhibiting bud activity (Doebley *et al*., 1997; Aguilar-Martínez *et al*., 2007; Martín-Trillo *et al*., 2011).

An alternative mechanism for the action of SL has been demonstrated, which strongly supports the canalisation-based model for bud regulation. A primary response to SL is the rapid depletion of the auxin transporter PIN1 from the plasma membrane, thus compromising canalisation of auxin transport from the bud into the main stem (Bennett *et al*., 2006; Crawford *et al*., 2010; Shinohara *et al*., 2013). This mode of action explains why, in several species, the addition of SL can only inhibit bud activity in the presence of a competing auxin source (Prusinkiewicz *et al*., 2009; Crawford *et al*., 2010; Liang *et al*., 2010; Ward *et al*., 2013), and why SL can sometimes promote bud activation (Liang *et al*., 2010; Shinohara *et al*., 2013; Ward *et al*., 2013).

Our understanding of the mechanism of action of SL depends heavily on mutants defective in SL synthesis and signalling. In contrast, most data on the role of CK in branching are based on monitoring the effects of the addition of CK on bud activity, and on correlations between CK levels or biosynthetic gene expression, auxin levels and bud activity (Cline, 1994; Turnbull *et al*., 1997; Bangerth *et al*., 2000; Nordström *et al*., 2004; Tanaka *et al*., 2006). Although these data support the second messenger hypothesis, it is clear from analysis of the action of SL that they are equally consistent with alternative explanations.

Genetic approaches to understanding the action of CK in shoot branching have been difficult, because many of the genes involved are present in large families (for review see Sakakibara, 2006; Hwang *et al*., 2012), and higher-order mutations exert pleiotropic effects, particularly on shoot growth and meristem activity (Higuchi *et al*., 2004; Leibfried *et al*., 2005; Miyawaki *et al*., 2006; Tokunaga *et al*., 2012). Here we describe a targeted approach to investigate the role of CK synthesis and response in shoot branching in Arabidopsis. Our results suggest that CKs play little part in auxin-mediated bud repression and release from apical dominance, but rather they provide a mechanism for buds to escape apical dominance and activate even in the presence of auxin.

## Results

### Isopentenyltransferases contribute to branching in intact Arabidopsis plants

Isopentenyltransferases (IPTs) catalyse an early step in CK biosynthesis, encoded by nine genes in Arabidopsis (Kakimoto, 2001; Takei *et al*., 2001a). *IPT1* and *IPT3–IPT8* are ATP/ADP IPTs and contribute to iP- and tZ-type CK synthesis (Miyawaki *et al*., 2006). As iP- and tZ-type CKs comprise the major CKs in Arabidopsis, these *IPT* genes were chosen for further investigation. *IPT4*,*IPT6* and *IPT8* are predominantly expressed in floral tissues, and are therefore unlikely to contribute to bud activation (Takei *et al*., 2001a; Miyawaki *et al*., 2004). The remaining four genes, *IPT1* (At1g68460), *IPT3* (At3g63110), *IPT5* (At5g19040) and *IPT7* (At3g23630), are expressed in roots and vegetative shoot tissues, and could be involved in shoot branching.

**Figure 1:**
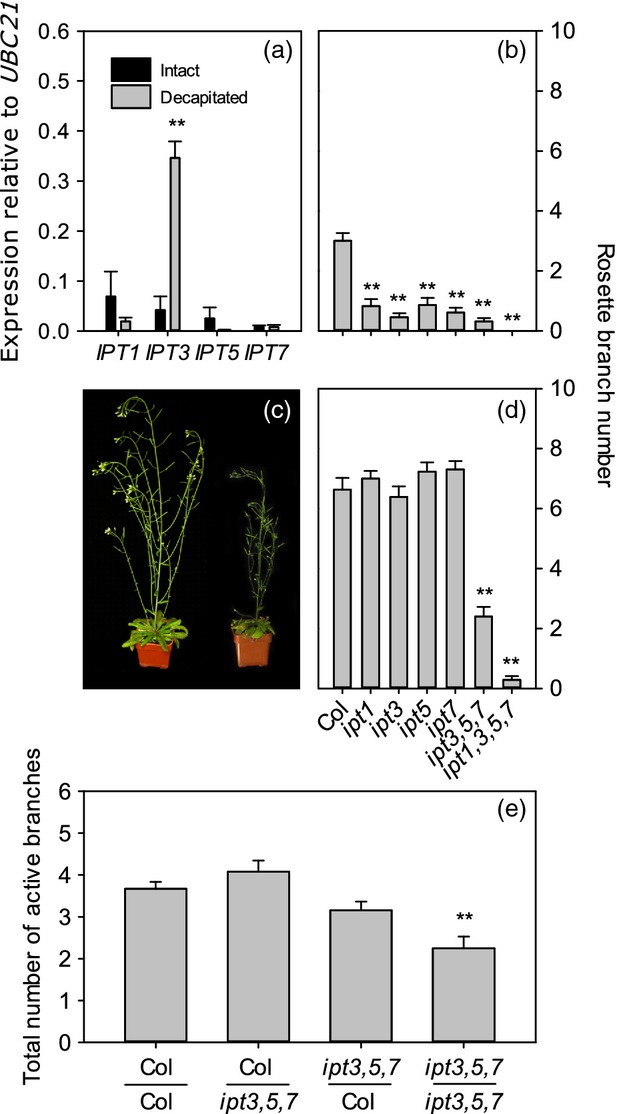
*IPT* gene expression and loss of function in intact, decapitated and grafted plants.(a) *IPT1*,*IPT3*,*IPT5* and *IPT7* transcript levels in isolated stems left intact or decapitated for 6 h. Mean expression levels (relative to *UBC21*) ± standard error (SE) are calculated from three biological replicates of 10–15 stems each.(b), (d) Rosette branch numbers in single triple and quadruple mutants of *ipt1*,*ipt3*,*ipt5* and *ipt7* in mature (6-week-old) plants left intact (b) or decapitated and analysed after one additional week (d). The mean branch number ± SE is shown (*n *=* *14–23).(c) Intact wild-type (left) and *ipt3,5,7* plants (right) at 6 weeks.(e) Total number of active branches (≥1 cm) in reciprocal shoot/root grafts between the wild type and *ipt3,5,7* at the onset of senescence (*n *=* *9–19).For (a) statistical comparisons were made between intact and decapitated samples using *t*-tests; asterisks denote a significance level of *P *<* *0.01 (**). For (b), (d) and (e) statistical comparisons were made between the wild type and each mutant using Mann–Whitney tests with a Bonferroni correction to adjust for multiple comparisons; asterisks (**) denote a significance level of *P *<* *0.0083 in (b) and (d), or *P *<* *0.0167 in (e).

In pea, CK is proposed to act as a second messenger for auxin because auxin in the PATS represses expression of *PsIPT1* and *PsIPT2*, whilst decapitation reduces stem auxin levels, resulting in increased *IPT* expression and levels of iP- and tZ-type CK in the stem (Tanaka *et al*., 2006). To test whether Arabidopsis *IPT* genes are similarly responsive to decapitation, the four selected *IPT* genes were assayed using an isolated two-node system (Ongaro *et al*., 2008; Prusinkiewicz *et al*., 2009). Two-node stem segments bearing the intact apex and inactive buds were collected and left for several days to allow the stem to elongate (Prusinkiewicz *et al*., 2009). The buds on these isolated segments grow out as normal if the apex is removed (Figure S1). Stem tissues were harvested 0 and 6 h after decapitation and expression levels of *IPT1*,*IPT3*,*IPT5* and *IPT7* determined relative to the reference gene *UBC21* using quantitative real-time PCR (qPCR) (Figure1a). Decapitation resulted in an eight-fold increase in *IPT3* compared with intact controls (*P *≤* *0.01), while the remaining *IPT* transcripts were not significantly affected.

Next, the effect of *IPT* loss-of-function on branching was investigated. Rosette branches were counted at maturity in single and higher-order mutant combinations of the four *IPT* genes (Miyawaki *et al*., 2006) (Figure1b). Single mutants showed significant reductions in branching: *ipt1*,*ipt3*,*ipt5* and *ipt7* all formed less than one branch on average (*P *<* *0.008 for all genotypes compared with the wild type). The *ipt3,5,7* mutant (Figure1c) formed a similarly low number of branches and *ipt1,3,5,7* formed no branches. This is consistent with the well-established role for CK in promoting branching.

We used grafting to test whether the *IPT* genes are required in the root or shoot to promote branching in intact plants. As *ipt1,3,5,7* is severely stunted, reciprocal grafts were performed between the *ipt3,5,7* triple mutant and the wild type. At maturity, self-grafted *ipt3,5,7* mutants possessed reduced branching compared to wild-type controls (*P *≤* *0.01), and the presence of either a wild-type root or shoot reciprocally grafted to *ipt3,5,7* was sufficient to confer wild-type levels of branching (Figure1e). Cytokinins produced in roots can therefore compensate for reduced CK synthesis in *ipt3,5,7* shoots, suggesting that CKs produced in the whole plant, rather than CKs produced in the shoot alone, contribute to branching in intact plants.

We next tested whether these *IPT* genes are required for auxin-mediated apical dominance by analysing the decapitation response of the *ipt* mutants. In particular, *ipt3* might have reduced branching as *IPT3* expression is responsive to decapitation (Figure1a), and *IPT* expression in the stem correlates with CK levels and bud outgrowth in pea (Tanaka *et al*., 2006). Mature plants were decapitated at the base of the bolt and rosette branch numbers were counted after 1 week (Figure1d). Surprisingly, most of the mutants, including *ipt3*, responded as the wild type, producing between 6.4 and 7.3 rosette branches. The exceptions to this were *ipt3,5,7* (2.4 ± 0.3 branches, *P *<* *0.008), and *ipt1,3,5,7* (0.3 ± 0.1, *P *<* *0.008).

**Figure 2:**
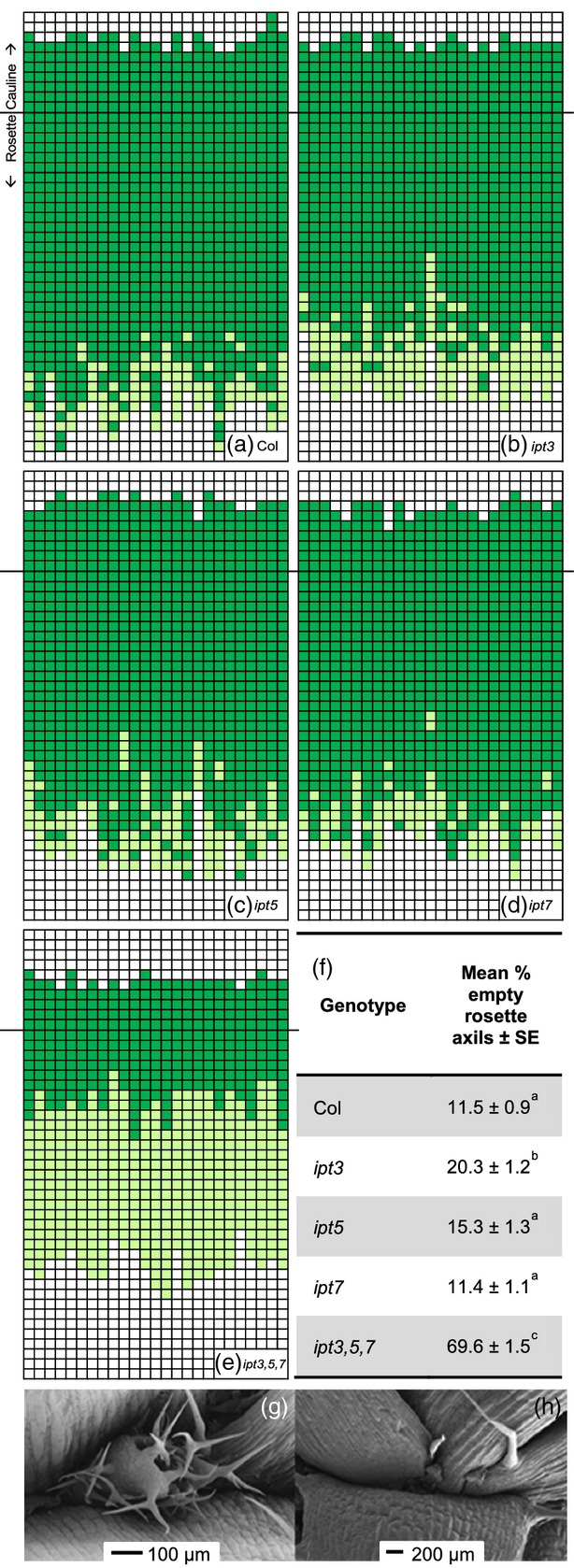
*ipt3,5,7* possesses fewer buds in its axils.Nodes with (dark green) or without (light green) a visible bud in (a) wild-type, (b) *ipt3*, (c) *ipt5*, (d) *ipt7* and (e) *ipt3,5,7* mutants (white represents no node). Each column represents an individual plant, aligned at the rosette–cauline transition. Cauline nodes are designated acropetally and rosette nodes basipetally. (f) The mean percentage of empty axils in the rosette ± SE (*n *=* *25). Statistical comparisons were performed using Tukey’s honestly significant difference test to assign homogeneous subsets. Scanning electron micrograph of (g) wild-type and (h) *ipt3,5,7* nodes.

Inspection of *ipt3,5,7* and *ipt1,3,5,7* showed that many of the axils lacked visible axillary buds, suggesting a failure in bud initiation, which would clearly preclude bud activation. We chose *ipt3,5,7* and the three cognate single mutants to quantify this axil phenotype in more detail. All genotypes were found to have some empty axils in more basal rosette nodes (Figure2a–e) when examined by eye. There was some evidence of an increased number of empty axils in *ipt3* (Figure2a,b), which had 20.3% empty axils compared with 11.5% in the wild type (*P *<* *0.001) (Figure2f). Both *ipt5* (Figure2c) and *ipt7* (Figure2d) had similar numbers of empty axils to the wild type. In *ipt3,5,7* the majority of rosette axils bore no visible bud (69.6%, *P *<* *0.001 compared with the wild type) (Figure2a,e). The reduction in decapitation-induced branching observed in *ipt3,5,7* (and *ipt1,3,5,7*) (Figure1c) is therefore probably due to compromised bud development. Consistent with this, scanning electron microscopy (SEM) revealed frequently empty axils in *ipt3,5,7* (Figure2g).

**Figure 3:**
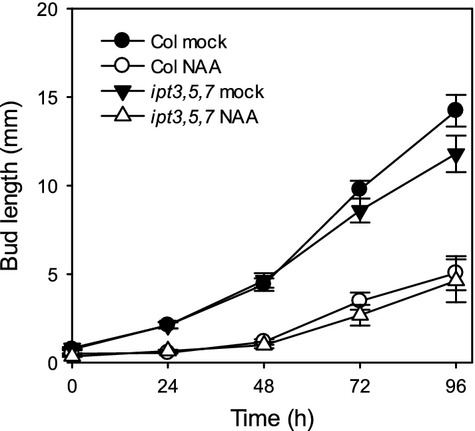
*ipt3,5,7* buds are responsive to apical auxin.Isolated nodal segments bearing one bud were treated apically for 4 days with 1 μmNAA or mock treatment. The mean bud length ± SE is shown (*n *=* *7–31). No statistically significant differences were found between genotypes over time in NAA treatments or mock treatments. Statistical comparisons were carried out using *t*-tests or Mann–Whitney tests.

Taken together, the *ipt* mutant analysis suggests that CK synthesis is required to allow high levels of branching in intact plants but not for activation of buds following decapitation. To confirm that *ipt3,5,7* buds are capable of responding to apical auxin, we investigated the response of *ipt3,5,7* buds to apical 1-naphthalene acetic acid (NAA) in an isolated nodal assay (Chatfield *et al*., 2000). The outgrowth of wild-type buds was delayed by apical auxin application as expected, and no significant differences were found between *ipt3,5,7* and the wild type over time in either treatment (Figure3). Together, these data suggest that *ipt3,5,7* buds can grow with wild-type kinetics and are auxin responsive, and therefore their reduced branching following decapitation (Figure1c) is likely to be due to defects in bud initiation.

These results suggest that the main role of CK in Arabidopsis branching is to allow the activation of buds in the presence of high auxin, thereby escaping apical dominance. Reduced CK synthesis, as in the *ipt* mutants, does not increase the sensitivity of buds to apical auxin, and high *IPT* activity is not required to activate buds following decapitation. These results are consistent with previous observations that CKs have relatively little effect on the outgrowth of isolated buds, but they can overcome the suppressive effect of apical auxin (Chatfield *et al*., 2000).

### Identification of auxin- and CK-responsive genes in buds

To investigate the effect of CK on buds inhibited by apical auxin, we compared the transcriptomes of mock-treated buds with those supplied with apical auxin alone or apical auxin combined with basal CK (6-benzylaminopurine, BA) in isolated nodes using the Affymetrix Arabidopsis gene chip ATH1. Buds were harvested after 18 h, allowing sufficient time for transcriptional changes to occur but before major size differences become apparent.

Comparison of the two treatments that activate buds, mock versus simultaneous auxin plus CK, revealed only two genes with statistically significant differences in expression: At1g75450, encoding a cytokinin oxidase (*CKX5*) (Schmülling *et al*., 2003; Werner *et al*., 2003), and At2g33830, a bud dormancy-associated gene (*DRM2*) (Tatematsu *et al*., 2005). Both genes were more highly expressed in buds treated with auxin plus CK than mock-treated buds.

To identify genes associated with bud activation, we focused on transcripts that were statistically significantly two-fold or more downregulated by apical auxin compared to mock-treated, but two-fold or more upregulated by simultaneous supply of basal CK compared to auxin alone. The number of genes meeting these criteria was 220 (Data S1). To select genes that might be particularly relevant for CK-mediated bud activation, we ranked them according to their upregulation in auxin plus CK compared with auxin alone. This produces a list in which the top 12 genes are upregulated five-fold or more (Table1). Of these 12, 11 have at least one *ARR1* response element (AAGATT) within 2000 bp upstream of their start codon, consistent with their CK responsiveness (Sakai *et al*., 2000, 2001; Taniguchi *et al*., 2007). Included in the 12 are *CKX5* and five members of a six-gene clade of type-A *ARR*s. The type-A *ARR*s are primary CK response genes, and are generally considered to be involved in feedback downregulation of CK signalling (Brandstatter and Kieber, 1998; Imamura *et al*., 1998; D’Agostino *et al*., 2000; To *et al*., 2004, 2007). This type-A *ARR* clade, comprising *ARR3* (At1g59940), *ARR4* (At1g10470), *ARR5* (At3g48100), *ARR6* (At5g62920), *ARR7* (At1g19050) and *ARR15* (At1g74890), was selected for further analysis of CK-mediated bud activation.

**Table 1:** The top 12 transcripts downregulated by apical auxin and upregulated by auxin and cytokinin (CK). All transcripts were classed as significantly (two-fold or more) decreased in auxin-treated buds compared with mock-treated buds, and increased five-fold or more in auxin and CK-treated buds compared with auxin-treated buds

Gene	ATH1 chip ID
1. At1g74890 (*ARR15,* ARABIDOPSIS RESPONSE REGULATOR 15)	262212_at
2. At1g19050 (*ARR7*, ARABIDOPSIS RESPONSE REGULATOR 7)	259466_at
3. At1g75450 (*CKX5*, CYTOKININ OXIDASE 5)	261109_at
4. At5g62920 (*ARR6*, ARABIDOPSIS RESPONSE REGULATOR 6)	247406_at
5. At1g03170 (*FAF2*, FANTASTIC FOUR 2)	264363_at
6. At3g30775 (*AtPOX*, PROLINE DEHYDROGENASE 1)	257315_at
7. At4g02810 (*FAF1*, FANTASTIC FOUR 1)	255448_at
8. At3g48100 (*ARR5*, ARABIDOPSIS RESPONSE REGULATOR 5)	252374_at
9. At5g19260 (*FAF3*, FANTASTIC FOUR 3)	249920_at
10. At3g62150 (*ABCB21*, ATP-BINDING CASSETTE B 21)	251248_at
11. At1g62480 (vacuolar calcium-binding protein-like protein)	265116_at
12. At1g10470 (*ARR4*, ARABIDOPSIS RESPONSE REGULATOR 4)	263236_at

### A clade of the type-A *ARR* family modulates intact branching patterns

We first verified the expression profile of the five *ARR* genes using qPCR. All genes were upregulated 2.5- to 11-fold in buds treated with basal CK and apical auxin compared with apical auxin alone (Figure S2).

**Figure 4:**
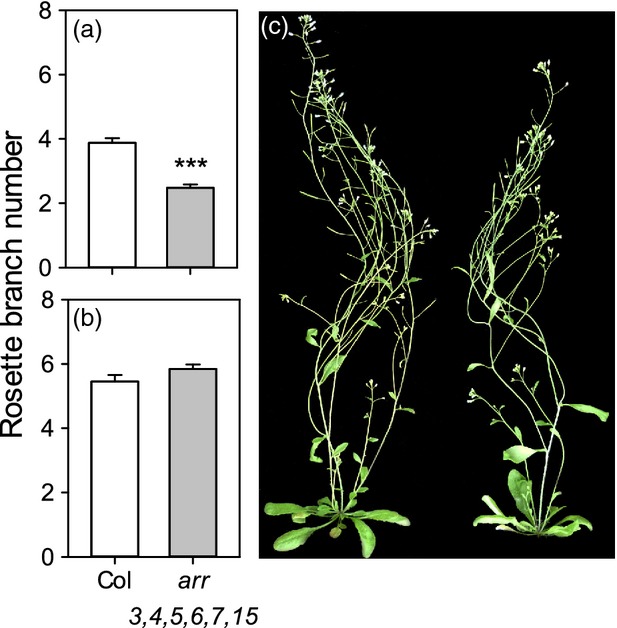
*arr3,4,5,6,7,15* has reduced branching in intact plants but a normal decapitation response.Rosette branches ≥0.5 cm were counted in (a) intact and (b) decapitated plants at maturity. The mean ± SE is shown (*n *=* *25–72). Statistical comparisons shown were made between the wild-type and *arr3,4,5,6,7,15* using the Mann–Whitney test. Asterisks denote a significance level of *P *<* *0.001 (***).(c) Intact wild-type (left) and *arr3,4,5,6,7,15* (right) plants at 5 weeks.

To investigate the role of this type-A *ARR* clade in bud activation, the hextuple *arr3,4,5,6,7,15* mutant branching phenotype was examined in mature plants left intact (Figure4a) or decapitated (Figure4b). Similar to *ipt* mutants, intact *arr3*,*4*,*5*,*6*,*7*,*15* plants formed significantly fewer rosette branches than the wild type (2.5 ± 0.1 versus 3.9 ± 0.1, respectively, *P *≤* *0.001) but there was no significant difference in decapitation-induced branching.

**Figure 5:**
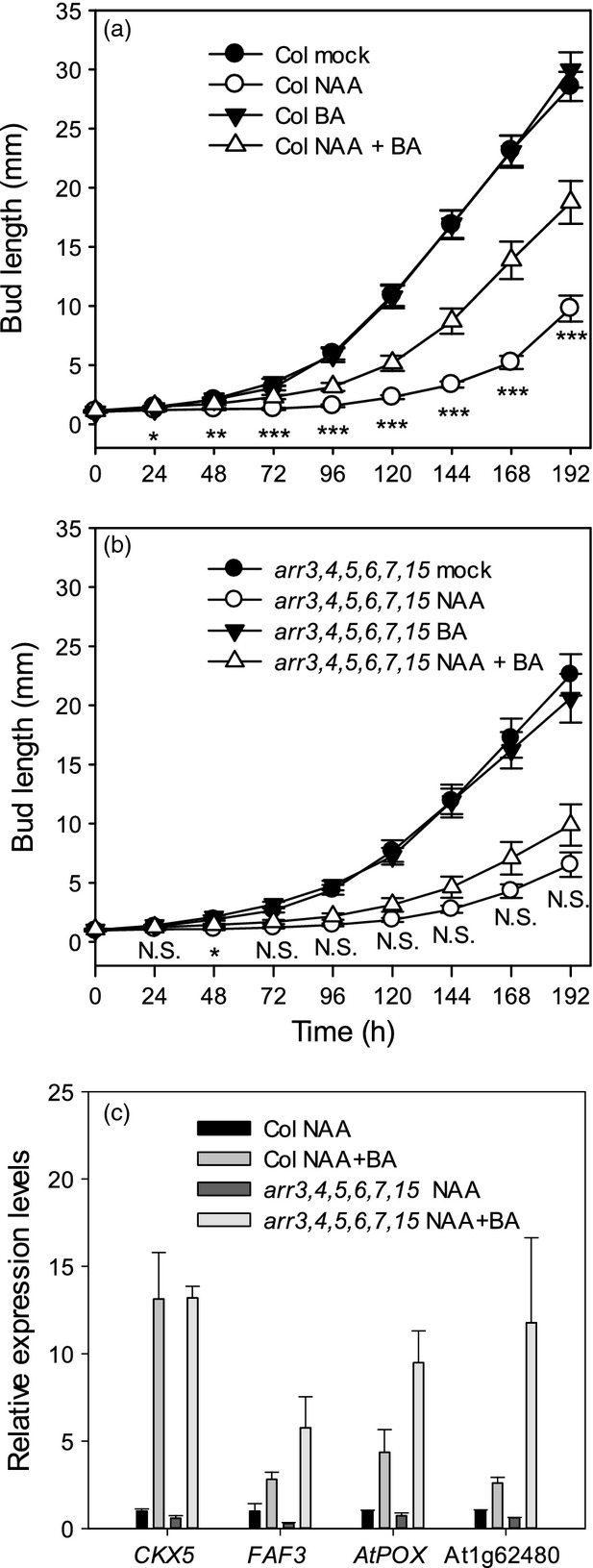
*arr3,4,5,6,7,15* buds have impaired outgrowth to CK but retain normal gene expression responses.(a) Wild-type and (b) *arr3,4,5,6,7,15* isolated nodal segments bearing one bud were treated for 8 days with mock, 1 μm NAA (apically), 1 μm 6-benzylaminopurine (BA; basally) or combined 1 μm NAA (apically) and 1 μm BA (basally). The mean ± SE is shown (*n *=* *20). Statistical comparisons shown were made between NAA and NAA + BA treated buds using *t*-tests and Mann–Whitney tests as the data did not always follow a normal distribution. Asterisks denote a significance level of *P *<* *0.05 (*), *P *<* *0.01 (**) or *P *<* *0.001 (***); N.S, not significant.(c) Expression of cytokinin-responsive genes in isolated buds treated with NAA and BA as in (a) and (b). Mean ± SE of two or three pools of 20 buds each is shown.

We used the isolated one-node assay to assess the CK response of *arr3*,*4*,*5*,*6*,*7*,*15* buds. Mock-treated mutant buds activated with normal kinetics and were inhibited by apical auxin, as for the wild type (Figure5). However, the ability of CK to overcome inhibition by apical auxin was compromised in *arr3*,*4*,*5*,*6*,*7*,*15*. Over time, there was no significant difference in length between mutant buds treated with apical auxin and those treated with apical auxin and basal CK, whereas wild-type buds treated with apical auxin and basal CK were significantly longer (*P *<* *0.01) than their apical-auxin-only equivalents from 48 h onward (Figure5b).

To determine whether transcriptional responses to CK are impaired in *arr3,4,5,6,7,15* buds, we used several genes from Table1 as markers and analysed their expression in wild-type and *arr3,4,5,6,7,15* buds treated with or without basal CK in the presence of apical auxin (Figure5c). Despite the fact that CK-treated wild-type buds activate while their *arr3,4,5,6,7,15* equivalents do not, CK treatment resulted in the upregulation of all the CK-responsive transcripts tested in the mutant buds at least as strongly as in wild-type buds.

Together, these results suggest that this type-A *ARR* clade is required for CK-mediated bud activation but not for bud transcriptional responses to CK. Furthermore, the results are consistent with the conclusion from the *IPT* gene analysis that CK is not required for release from auxin-mediated apical dominance, but rather acts to overcome the inhibitory effects of apical auxin, promoting branching in intact plants.

### Strigolactone does not rescue decapitation-induced bud outgrowth in CK mutants

**Figure 6:**
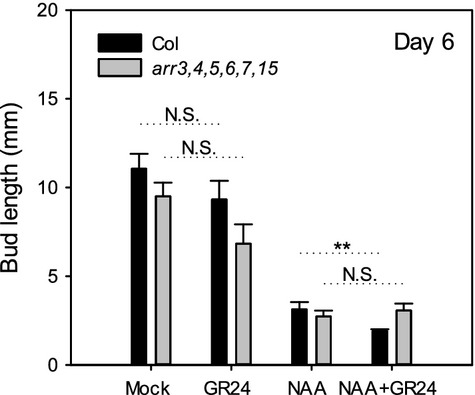
Strigolactone does not inhibit *arr3,4,5,6,7,15* bud outgrowth.(a) Wild-type and (b) *arr3,4,5,6,7,15* isolated nodal segments bearing one bud were treated with mock, 0.5 μm NAA (apically), 5 μm GR24 (basally) or combined 0.5 μm NAA and 5 μm GR24 (basally). The mean ± SE on day 6 is shown (*n *=* *20). Statistical comparisons shown were made using *t*-tests or Mann–Whitney tests as the data did not always follow a normal. Asterisks denote a significance level of *P *<* *0.01 (**); N.S., not significant.

It has previously been suggested that bud activity represents a read-out of the ratio of CK to SL in buds, with both being influenced by auxin in the main stem (Dun *et al*., 2012). This may explain why in many species SL is unable to inhibit buds in the absence of a competing auxin source, as high CK levels resulting from low auxin might render the buds resistant to SL. According to this hypothesis, *arr3,4,5,6,7,15* buds may respond normally to decapitation despite their lack of CK response because of low levels of SL. If this is the case, isolated *arr3,4,5,6,7,15* buds should fail to activate in the presence of high SL. We therefore tested the response of *arr3,4,5,6,7,15* buds to the synthetic SL GR24 (Figure6). As previously described by Crawford *et al*. (2010), wild-type buds activated similarly with or without basal SL, and SL applied in combination with apical auxin inhibited buds to a greater extent than auxin alone (*P *≤* *0.01). *arr3,4,5,6,7,15* buds responded similarly to the wild type. There was a slight reduction in SL-treated *arr3,4,5,6,7,15* bud elongation relative to mock, but this was not statistically significant. Additionally, *arr3,4,5,6,7,15* buds exhibited some resistance to SL, as there was no effect of SL on bud activation in the presence of apical auxin. The finding that wild-type and *arr3,4,5,6,7,15* buds alike activate under high SL suggests that depletion of auxin in the main stem alone is sufficient to trigger sustained bud activity, with no requirement for low SL and/or high CK, consistent with the canalisation-based hypothesis for bud activation.

### Branching response to high nitrate is impaired in higher-order CK mutants

**Figure 7:**
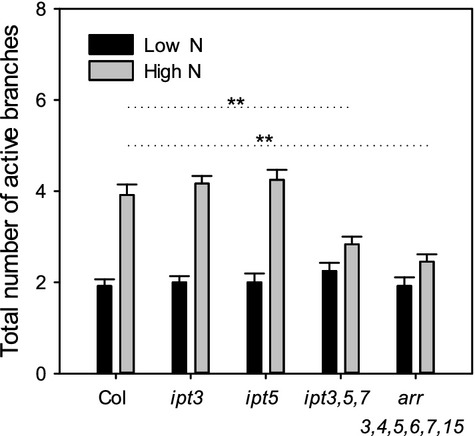
The branching plasticity response to high N is reduced in higher-order cytokinin mutants.Plants were grown on soil-free medium under high (9 mm NO_3_) or low (1.8 mm NO_3_) nitrate conditions and the total number of active rosette and cauline branches (≥1 cm) counted at the onset of senescence. The mean ± SE is shown (*n *=* *11–12). The statistical comparisons shown were made between the wild type and each mutant on high nitrate using Mann–Whitney tests with a Bonferroni correction to adjust for multiple comparisons; asterisks denote a significance level of *P *<* *0.0125 (**). No significant differences were found between the wild type-and *ipt3* or *ipt5* on high nitrate or between the wild type and any mutants on low nitrate.

Our data support the hypothesis that the main role of CK in branching is to drive bud activation in the presence of high auxin. One situation where this might be physiologically relevant is during changes in nitrate availability. Low nitrate availability is known to increase in the proportion of biomass allocated to roots versus shoots (Drew, 1975; Scheible *et al*., 1997) and high N is associated with bud activation (McIntyre and Hunter, 1975; McIntyre and Cessna, 1991; Ding *et al*., 1995; McIntyre, 2001; Liu *et al*., 2011; de Jong *et al*., 2014). As nitrate is known to promote CK synthesis (Takei *et al*., 2001b, 2002, 2004), CK might drive the activation of additional shoot branches despite the high main stem auxin contributed by already active shoots. To test this, we compared total branch numbers of CK mutants with the wild-type under high- and low-nitrate conditions (Figure7). Although *ipt3* and *ipt5* single mutants have similar branching levels to the wild type when grown under these conditions, the *ipt3,5,7* and *arr3,4,5,6,7,15* mutants have significantly fewer active branches than the wild type on high nitrate. In contrast, there was no difference between the wild type and the mutants on low nitrate. These data suggest that CK is not required to support branching on low nitrate but that it may be important to enhance branching under high nitrate.

## Discussion

Many of the genes involved in CK biosynthesis and signalling are members of large families, bringing the combined problems of functional redundancy and pleiotropy to mutant analyses. To investigate the role of CK in shoot branching in Arabidopsis we selected mutants of only the most relevant members of the *IPT* family of CK biosynthetic genes and the *ARR* family of CK signalling genes based on transcriptional profiles. The fact that we observed similar mutant phenotypes for the selected *IPT* and *ARR* genes suggests that this strategy was effective, though some differences were observed. For example, our results support a role for *IPT*-mediated CK synthesis in bud initiation (Figure2; Wang *et al*., 2014) that apparently does not require the *ARR*s involved in bud activation.

Type-A *ARR* multiple mutants have CK-response phenotypes in roots (To *et al*., 2004, 2007; Zhang *et al*., 2011). It is therefore possible that some of the effects observed in shoots are influenced by pleiotropy. Furthermore, it cannot be excluded that other family members may play roles in bud activation, for example rarer CK types dependent on *IPT2* and *IPT9* may be involved. Nonetheless, the combined dataset demonstrates the relatively direct effects of CK on bud activation, and that CK synthesis and signalling contribute to branching in Arabidopsis. Particularly compelling in this regard are the wild-type activation and growth kinetics of the CK-related mutant buds in response to decapitation, demonstrating the general vigour of the buds, and the CK resistance of *arr* hextuple mutant buds in isolated nodal stem segments, where there are no roots.

### The role of CK in auxin-mediated apical dominance

Our results in Arabidopsis are consistent with a substantial body of evidence that CK promotes bud outgrowth in many species. This, in combination with correlative studies, has led to the hypothesis that auxin inhibits bud outgrowth by restricting the supply of CK to buds. This hypothesis is well supported in pea by the transcriptional responses of two *IPT* genes to decapitation and auxin supply, and changes in CK levels in the stem (Tanaka *et al*., 2006; Shimizu-Sato *et al*., 2009). Auxin repression of stem *IPT* gene expression is conserved across species, for example in chrysanthemum (Chen *et al*., 2013) and rice (Minakuchi *et al*., 2010). We found similar results in Arabidopsis, but only for *IPT3* (Figure1a). Interestingly, these results contrast with those in roots, where auxin has little effect on *IPT3* but upregulates *IPT5* and *IPT7* (Miyawaki *et al*., 2004). The auxin responsiveness of different *IPT* family members may therefore be determined by tissue-specific factors.

The transcriptional response of *IPT3* to decapitation is consistent with the idea that auxin-mediated bud inhibition involves a reduced supply of CK. Similarly, our observation that buds activated by decapitation have virtually identical transcriptomes to buds activated by basal CK supply in the presence of apical auxin could reflect an important role for high CK in bud activation following decapitation. However, our mutant analysis does not support this hypothesis. Multiple *ipt* mutant buds were unaffected in their response to apical auxin or to presumed auxin depletion in the main stem PATS following decapitation. Furthermore, *arr3*,*4*,*5*,*6*,*7*,*15* buds, despite being severely compromised in CK-mediated bud activation, are fully capable of responding to apical auxin and decapitation. In Arabidopsis it therefore seems that CK is not required for release from auxin-mediated apical dominance following decapitation, but rather it allows buds to escape apical dominance and activate even when auxin in the PATS is high.

In pea it has been suggested that bud activity is determined by the ratio of CK and SL in buds (Braun *et al*., 2012; Dun *et al*., 2012). Auxin-mediated changes in SL levels could explain the normal activation of *arr3*,*4*,*5*,*6*,*7*,*15* mutant buds following decapitation (Figure4b) and their inhibition by apical auxin (Figure5b). However, this seems unlikely because SL is unable to prevent the activation of *arr3*,*4*,*5*,*6*,*7*,*15* buds on isolated nodes (Figure6). Our results from Arabidopsis are therefore not consistent with CK:SL ratios regulating bud activity, highlighting the need to better understand the mechanism by which CK activates buds.

### Transcriptional CK responses in buds

There are several hypotheses about how CK promotes bud outgrowth (for a review see Müller and Leyser, 2011). Most involve CK-induced transcription of genes such as those involved in cell-cycle regulation or meristem function, and there is good evidence that the CK signalling network regulates the transcription of such genes (Riou-Khamlichi *et al*., 1999; Suzuki *et al*., 2001; Dewitte *et al*., 2007; Braun *et al*., 2012; Dun *et al*., 2012).

We identified 220 genes that are significantly (two-fold or more) downregulated by apical auxin and also significantly (two-fold or more) upregulated by simultaneous basal CK supply (Table S1). This includes several type-A *ARR*s and CK metabolism genes, which have been repeatedly identified as CK-responsive in meta-analyses of CK microarrays (Brenner *et al*., 2012; Bhargava *et al*., 2013). The list also includes several genes involved in DNA replication, but not classical CK cell-cycle targets such as cyclin D3 (Riou-Khamlichi *et al*., 1999) or known CK-regulated meristem regulators such as *WUS* (Lindsay *et al*., 2006; Gordon *et al*., 2009). However, the list does include three members of the FANTASTIC FOUR (*FAF*) family, of which *FAF2* has been implicated in regulating shoot meristem size. Over-expression of *FAF*s results in reduced meristem size and reduced *WUS* expression, and *FAF2* is expressed in the *WUS* domain of the shoot apical meristem (Wahl *et al*., 2010). *FAF1* and *FAF3* can also influence meristem size when ectopically expressed, but their native expression pattern is primarily in the vasculature. *FAF3* has previously been identified as robustly inducible by CK (Bhargava *et al*., 2013).

An additional hypothesis for the mechanisms of action of CK in bud activation is promotion of auxin export from buds. This could be at the transcriptional level, and, consistent with this hypothesis, several genes involved in auxin biosynthesis and auxin transport are upregulated in the auxin plus CK treatment. This includes the auxin transporter ABCB21 (Table1) (Kamimoto *et al*., 2012).

### The role of type-A *ARR*s in bud activation

To assess the role of the upregulated type-A *ARR*s in the control of bud growth we analysed a hextuple mutant lacking all members of the upregulated clade. The mutant buds were strongly resistant to activation by basally applied CK, consistent with their reduced branching phenotype in intact plants. The inability of these buds to activate in response to CK raises interesting questions about the mechanism of action of CK in promoting bud growth. The hextuple mutant buds are apparently fully responsive to CK at the transcriptional level (Figure5c), suggesting that these transcriptional changes are insufficient to activate buds in the absence of the type-A *ARR* clade. Given the reduced branching phenotype of the mutant, but normal growth kinetics in active branches, a likely explanation is that there are essential type-A *ARR*-dependent non-transcriptional roles for CK in bud activation.

### Post-transcriptional CK responses

In roots, CK reduces the abundance of some PIN auxin efflux carriers at the plasma membrane via a post-transcriptional mechanism (Marhavý *et al*., 2011; Zhang *et al*., 2011). For PIN1, this effect is dependent on AHK4 and two type-B ARRs (Marhavý *et al*., 2011). Type-A *arr* mutants are hypersensitive in their PIN responses to CK, suggesting that they function as negative regulators of CK with respect to this non-transcriptional pathway (Zhang *et al*., 2011). In light of this our results appear paradoxical, because loss of multiple type-A *ARR*s should lead to hypersensitivity to CK yet *arr3,4,5,6,7,15* buds exhibit CK resistance and intact *arr3,4,5,6,7,15* plants have reduced branching, similar to the loss of certain *IPT* CK biosynthetic genes.

This paradox can be resolved if the primary target for CK signalling in bud regulation is plasma membrane accumulation of PIN proteins, and bud activation requires canalisation of auxin transport from the bud to the stem, as proposed in the canalisation-based model for branching control. In roots, there is some evidence of differential sensitivity to CK of PINs on different cell faces (Marhavý *et al*., 2014). If this is also the case for shoots, then low levels of CK might promote the establishment of auxin transport canalisation out of buds by removing PINs differentially from non-rootward cell faces. If *arr3,4,5,6,7,15* buds and/or shoots are hypersensitive to CK this could result in broader PIN removal, including rootward PINs, reducing the ability to canalise auxin transport out of the bud. This idea is highly speculative, and it certainly cannot be assumed that the behaviour of PIN in roots and buds will be the same; however, it does provide a possible explanation for the molecular and physiological phenotypes of the *arr3,4,5,6,7,15* mutants, which can be tested at a cellular level.

### The response of CK and branching to the environment

Our data strongly suggest that in Arabidopsis CK drives increased bud activation when auxin levels are high. This solves a major problem with apical dominance as a mechanism for modulating shoot architecture. Under this model, if a vigorous apex exports auxin and inhibits the buds below it, apical dominance would be strong under favourable conditions, restricting the plant’s capacity to produce branches despite resource abundance. A canalisation-based mechanism for apical dominance is less problematic, because in this case bud activation is essentially a competitive process; conditions that increase the vigour of the primary apex will also increase the vigour of lateral buds. The mode of action for CK suggested by our data allows for the exploitation of favourable conditions through CK-mediated escape from apical dominance. There is evidence that CK synthesis is increased by high nitrate (for review, see Kiba *et al*., 2011) at least partly via the upregulation of *IPT* gene expression (Miyawaki *et al*., 2004; Takei *et al*., 2004), and that CK can act as a systemic signal for nitrate status (Ruffel *et al*., 2011), consistent with our grafting results (Figure1e). Our findings suggest that an N-induced increase in CK contributes to increased branching because both *ipt3,5,7* and *arr3*,*4*,*5*,*6*,*7*,*15* have wild-type-levels of branching when grown under low-nitrate conditions, but they are compromised in their ability to activate more buds when nitrate levels are higher (Figure7). In the case of *ipt3,5,7,* this could be partly due to bud initiation defects, but this is not the case for *arr3*,*4*,*5*,*6*,*7*,*15*.

Further consideration of this mechanism also provides a possible function for the downregulation of CK in the stem by auxin. If unchecked, escape from auxin-mediated apical dominance could lead to the activation of all buds. This could be prevented by downregulation of CK biosynthesis by additional auxin, supplied by the newly activated branches. Thus, auxin and CK can act in a feedback loop, the equilibrium of which can be adjusted by auxin-independent CK synthesis driven by external inputs such as nutrient availability or light.

## Experimental Procedures

### Plant lines and growth conditions

Col-0 was used as the wild-type controls and all mutant lines were on a Col-0 background. Seeds were stratified for 3–5 days at 4°C. The *ipt1-1*,*ipt3*-*2*,*ipt5-2* and *ipt7-1* single, double, triple and quadruple mutant combinations used were those described in Miyawaki *et al*. (2006). The *arr* alleles are those previously described in Zhang *et al*. (2011). All soil-grown plants were sown on F2 compost in P40 trays treated with Intercept 70WG (both Levington, http://www.scottsprofessional.co.uk) then transferred to the glasshouse or controlled environment rooms. Glasshouse conditions comprised a long-day photoperiod (16-h light/8-h dark) and an average temperature range of 15–25°C. Controlled environment conditions comprised either long-day or short-day (8-h light/16-h dark) photoperiods, an average light intensity of 170 or 100 μmol m^−2^ sec^−1^ for soil-grown and sterile-grown plants, respectively, and an average temperature range of 17–21°C.

### Statistical analyses

Based on the assumption that branch numbers do not follow a normal distribution, the Mann–Whitney test was used for statistical comparisons of non-parametric data and a Bonferroni correction applied for multiple comparisons. Kolmogorov–Smirnov and Shapiro–Wilk tests for normality were used to determine if other datasets followed a normal distribution. For parametric distributions, statistical analyses were performed using two-tailed *t*-tests and interpreted using Levene’s test for homogeneity of variance.

### Two-node assays

Two-node assays (Figures1a and S1) were performed as described in Ongaro *et al*. (2008) and Prusinkiewicz *et al*. (2009).

### Intact and decapitated branch counts

For *ipt* mutant branch counts (Figure1b,c), plants were soil-grown under short days in controlled-environment rooms for 6 weeks then transferred to long-day glasshouse conditions. After flowering, plants were left intact or decapitated at the base of the bolt and rosette branch numbers ≥0.5 cm recorded after 1 week. For *arr3,4,5,6,7,15* mutant branch counts (Figure5a), intact plants were grown under glasshouse conditions and the number of branches ≥0.5 cm were recorded when plants had developed at least two full siliques. The decapitation assays shown in Figure5(b) were performed as per Greb *et al*. (2003).

### Grafting

Reciprocal grafts between the wild type and *ipt3,5,7* (Figure1e) were performed using the transverse cut and butt alignment method as per Turnbull *et al*. (2002) with the following modifications. Seeds were germinated on *Arabidopsis thaliana* salts (ATS) medium (Wilson *et al*., 1990) containing 0.8% bacto-agar and grown on vertically mounted Petri dishes under sterile short-day controlled-environment conditions as outlined above. Seven-day-old seedlings were used for grafting and 7–9 days after grafting, plants showing root growth and no adventitious roots were transferred to sand and Terra-Green on a high-nitrate regime as outlined below. The total number of active rosette and cauline branches (≥1 cm) were counted at the onset of senescence when the oldest siliques had ripened.

### *ipt* axil characterisation

Axils were examined by eye via light microscopy (Figure2a–f) and buds classified as present if at least one bud leaf was discernible. For SEM (Figure2g,h), plants were soil-grown for 6 weeks under short-day conditions in controlled environment rooms as described. Emerging bolts, roots and rosette leaf blades were removed and the remaining rosette stems fixed with 4% paraformaldehyde, washed in phosphate buffer then dehydrated in an acetone series up to 100%. Critical point drying was performed at the York University Technology Facility. Specimens were subsequently coated with gold/palladium and imaged using a JEOL JSM-6490LV microscope (http://www.jeol.co.jp/en/).

### One-node assays

Plants were grown under sterile long-day conditions as described in Bennett *et al*. (2006). Cauline stem segments bearing the basal inactive bud were assayed as per Chatfield *et al*. (2000). For CK treatment, BA (Sigma, http://www.sigma.com) was dissolved in DMSO and applied basally as a 1000× stock to a final concentration of 1 μm. For auxin treatment, NAA (Sigma) was dissolved in 70% ethanol and applied apically as a 1000× stock to a final concentration of 0.5 or 1 μm. GR24 (LeadGen Labs, http://www.leadgenlabs.com/) was dissolved in acetone and applied as a 1000× stock to a final concentration of 5 μm. For mock treatments, DMSO or 70% ethanol was applied basally or apically, respectively, at 0.1% v/v.

### Microarray study

One-node segments were treated with apical NAA alone, apical NAA and basal BA or mock controls as described. Buds were harvested onto liquid nitrogen after 18 h. Total RNA was extracted using the RNeasy Micro Kit (Qiagen; http://www.qiagen.com/). For each microarray, 300 ng total RNA input was used. The RNA labelling and preparation for hybridisation was carried out using an Ambion MessageAmp III aRNA amplification kit (http://www.lifetechnologies.com/) according to the manufacturer’s instructions. An Affymetrix GeneChip Poly-A RNA control kit was used for controls (http://www.affymetrix.com/). The Affymetrix Arabidopsis gene chip ATH1 was hybridised. Three biological replicates were analysed. Statistical analyses of microarray data were performed using R software version 2.15 (http://www.r-project.org/) and the Affy (Gautier *et al*., 2004), Affycoretools and Bioconductor Limma packages. Raw data were normalised using the robust multichip analysis (RMA) algorithm. Differential gene expression between groups was then determined by fitting a linear model to the data using lmfit with subsequent comparisons made using the makeContrasts function. Transcripts with a *q*-value (Hochberg and Benjamini, 1990) of less than 0.05 and a fold change of two or more were classed as significantly differentially regulated. Microarray data were deposited at Gene Expression Omnibus (http://www.ncbi.nlm.nih.gov/geo/), accession number GSE59741.

### Quantitative real-time PCR

For *IPT* gene expression analysis (Figure1a), two-node assays were set up as described and left intact for 4–6 days. Only segments bearing inactive buds were used. Plants were left intact or decapitated at the apex for 6 h, nodal stems (with axillary buds removed) harvested onto liquid nitrogen or RNAlater (Ambion, http://www.invitrogen.com/) into three pools of 10–15 segments each. Total RNA was extracted using the RNeasy Plant Mini kit (Qiagen) and subjected to DNAse treatment using the Turbo DNA-free kit (Ambion) as per the manufacturer’s instructions. The RNA was quantified using a NanoDrop 1000. For cDNA synthesis, 1 μg of total RNA was reverse transcribed with Superscript II (Invitrogen, http://www.invitrogen.com/) according to the manufacturer’s instructions. Quantification of transcript levels was carried out using SYBR Green reactions with 5 ng cDNA in a 20 μl volume on a Light Cycler 480 II (Roche, http://www.roche.com/) relative to the reference gene UBIQUITIN-CONJUGATING ENZYME 21 (*UBC21*, At5g25760). Three technical replicates were run for each biological replicate and averaged. Means shown represent the average of biological replicates. Expression levels were calculated using the Light Cycler 480 II software and the second-derivative maximum method assuming equal primer efficiencies.

### Nitrate response

Seeds were sown in 5-cm plastic pots on a 1:1 mix of Leighton Buzzard sand (WBB Minerals, http://www.wbbminerals.net/) and Terra-Green (Oil-Dri, http://www.oil-dri.co.uk) fed with 25 ml nitrate-sufficient ATS medium (containing 9 mm NO_3_; Wilson *et al*., 1990) or nitrate-insufficient ATS [containing 1.8 mm NO_3_, made by adjusting the following ATS components and their final concentrations: 0.4 mm Ca(NO_3_)_2_, 1 mm KNO_3_, 4 mm KCl and 1.6 mm CaCl_2_] and grown under glasshouse conditions. After 2 weeks, pots were fed weekly with 10 ml of nutrient solution. Branch numbers were scored as outlined above for grafted plants.

### Additional experimental procedures

Additional experimental procedures are described in Methods S1.

## Supporting Information

**Figure S1.** Decapitation response of buds in an isolated two-node assay.

**Figure S2.** Quantitative real-time PCR confirmation of *ARR* gene expression in buds treated with basal cytokinin and apical auxin.

**Table S1.** Quantitative real-time PCR primer sequences

**Data S1.** Transcripts downregulated by apical auxin and upregulation by auxin and cytokinin.

**Methods S1.** Supporting experimental procedures.
