# Supplementary material for: Cytokinin is required for escape but not release from auxin mediated apical dominance
Source: Plant J. 2015 May 12;82(5):874–86. doi: 10.1111/tpj.12862 (PMC4691322; doi:10.1111/tpj.12862)
Supplement: Supplementary file 1 [file tpj0082-0874-sd1.pdf]

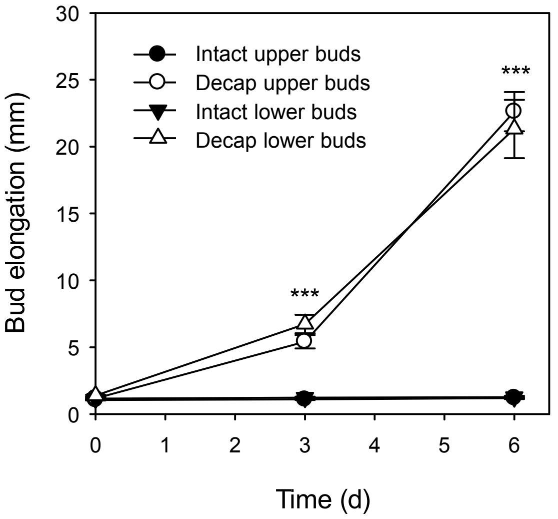

Figure S1. Decapitation response of wild-type buds in an isolated two-node assay. Stem segments bearing two nodes and the apex were set up as per Ongaro *et al.* (2008) and kept intact for 4-6 days. Subsequently, the segments were left intact or decapitated and bud lengths measured over time for a further 6 days. Mean ± SE are shown (n = 17-20). Statistical comparisons were made between intact and decapitated buds at each time point using t-tests. Asterisks denote a significance level of $p < 0.001$ (***) for both upper and lower buds.
