# Supplementary material for: Cytokinin is required for escape but not release from auxin mediated apical dominance
Source: Plant J. 2015 May 12;82(5):874–86. doi: 10.1111/tpj.12862 (PMC4691322; doi:10.1111/tpj.12862)
Supplement: Supplementary file 2 [file tpj0082-0874-sd2.pdf]

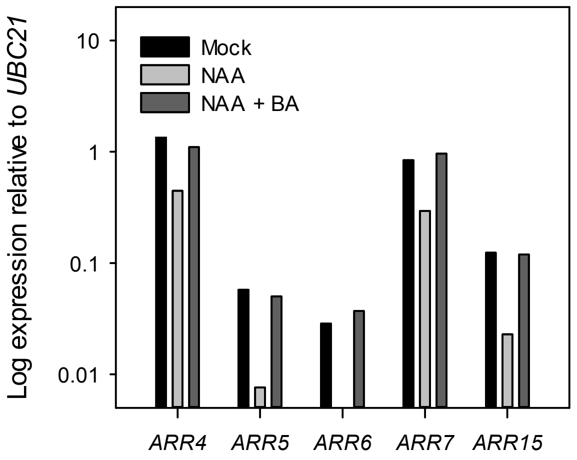

Figure S2. qPCR confirmation of *ARR* gene expression in buds treated with basal CK and apical auxin. Isolated nodal segments bearing one bud were treated for 18 h with hormone control (0.1% v/v 70 % ethanol apically and 0.1% v/v DMSO basally), NAA alone (1 µM NAA apically and 0.1% v/v DMSO basally) or NAA + BA (1 µM NAA apically and 1 µM BA basally). The mean of a single pool of 20 buds is shown.
