# Supplementary material for: Cytokinin is required for escape but not release from auxin mediated apical dominance
Source: Plant J. 2015 May 12;82(5):874–86. doi: 10.1111/tpj.12862 (PMC4691322; doi:10.1111/tpj.12862)
Supplement: Supplementary file 3 [file tpj0082-0874-sd3.docx]

**Table S1.** qPCR primer sequences

| **Primer** | **Sequence (5’-3’)** |
| --- | --- |
| *IPT1* forward | CGATTATCGCCGGTGGATCT |
| *IPT1* reverse | CGTAACGCAAATCGGAGCTG |
| *IPT3* forward | AGACTTCCCTCCAGCGAGAT |
| *IPT3* reverse | GAGAGTCGTGACTTGCCTGT |
| *IPT5* forward | ACCTAGCCACTCGTTTTCCG |
| *IPT5* reverse | TTCGTAAGTGTCGTGGACGG |
| *IPT7* forward | CCTCACCACTTGCTTGGAGT |
| *IPT7* reverse | GGGAAGCTTGTTGTTCGCTG |
| *CKX5* forward | CTCGACGTTGTAACTGGGAAA |
| *CKX5* reverse | GCTGGTTCGAGAGAGATTCGT |
| *FAF3* forward | As per ([Wahl *et al.,* 2010](#_ENREF_89)) |
| *FAF3* reverse | As per ([Wahl *et al.,* 2010](#_ENREF_89)) |
| *AtPOX* forward | GGATTCCCTGGGTTGCAAGT |
| *AtPOX* reverse | GACGCAAGTCTCCCCGAATC |
| At1g62480 forward | GGCCACCATCGAGGTTGAA |
| At1g62480 reverse | TTGTTACTTCGACAGGCGCT |
| *ARR4* forward | GACCAGAATCGACAGATGCCT |
| *ARR4* reverse | AGCTTCCGTTTGTTTCCGTT |
| *ARR5* forward | TGTCGATAGTGCGACAAGAGC |
| *ARR5* reverse | CTTCAGATCCTCAAATCCAACCG |
| *ARR6* forward | TCGAGCGTTTGCTCAGAGTAT |
| *ARR6* reverse | AACCTTCAAATCCTCAAAACCGA |
| *ARR7* forward | TCAATGCCAGGACTTTCAGGA |
| *ARR7* reverse | TGCTCCTTCTTTGAGACATTCTTG |
| *ARR15* forward | TCAATGCCGGGACTAACAGG |
| *ARR15* reverse | TGCTCCTTCTATCATACATTGTTCT |
| *UBC21* forward | TCCTCTTAACTGCGACTCAGG |
| *UBC21* reverse | GCGAGGCGTGTATACATTTG |

**Data S1.** Transcripts down-regulated by apical auxin and up-regulated by auxin and CK.
