# Supplementary material for: Cytokinin is required for escape but not release from auxin mediated apical dominance
Source: Plant J. 2015 May 12;82(5):874–86. doi: 10.1111/tpj.12862 (PMC4691322; doi:10.1111/tpj.12862)
Supplement: Supplementary file 5 [file tpj0082-0874-sd5.docx]

**Methods S1: Supporting experimental procedures**

**qPCR confirmation of microarray results**

For *ARR* gene expression analysis (Figure S2), one-node assays were set up as described for the microarray study. RNA extractions, cDNA synthesis and qPCR were performed as described for the *IPT* genes except that a single pool was collected and analysed. qPCR primer sequences are listed in Table S2.
