# Supplementary material for: Cytokinin is required for escape but not release from auxin mediated apical dominance
Source: Plant J. 2015 May 12;82(5):874–86. doi: 10.1111/tpj.12862 (PMC4691322; doi:10.1111/tpj.12862)
Supplement: Supplementary file 6 [file tpj0082-0874-sd6.docx]

**Figure S1.** Decapitation response of wild-type buds in an isolated two-node assay. Stem segments bearing two nodes and the apex were set up as per Ongaro *et al.* ([2008](#_ENREF_57)) and kept intact for 4-6 days. Subsequently, the segments were left intact or decapitated and bud lengths measured over time for a further 6 days. The mean ± SE are shown (*n* = 17-20). Statistical comparisons were made between intact and decapitated buds at each time point using *t*-tests. Asterisks denote a significance level of *p* < 0.001 (***) for both upper and lower buds.

**Figure S2.** qPCR confirmation of *ARR* gene expression in buds treated with basal CK and apical auxin. Isolated nodal segments bearing one bud were treated for 18 h with hormone control (0.1% v/v 70 % ethanol apically and 0.1% v/v DMSO basally), NAA alone (1 µM NAA apically and 0.1% v/v DMSO basally) or NAA + BA (1 µM NAA apically and 1 µM BA basally). The mean of a single pool of 20 buds is shown.

**Table S1.** qPCR primer sequences

**Data S1.** Transcripts down-regulated by apical auxin and up-regulated by auxin and CK.

**Methods S1:** Supporting experimental procedures.
